# Collagen co-localized with macrovesicular steatosis better differentiates fibrosis progression in non-alcoholic fatty liver disease mouse models

**DOI:** 10.3389/fmed.2023.1172058

**Published:** 2023-06-02

**Authors:** Xiao-Xiao Wang, Rui Jin, Xiao-He Li, Qiang Yang, Xiao Teng, Fang-Fang Liu, Nan Wu, Hui-Ying Rao, Feng Liu

**Affiliations:** ^1^Peking University People’s Hospital, Peking University Hepatology Institute, Beijing Key Laboratory of Hepatitis C and Immunotherapy for Liver Diseases, Beijing International Cooperation Base for Science and Technology on NAFLD Diagnosis, Beijing, China; ^2^Hangzhou Choutu Technology Co., Ltd., Hangzhou, China; ^3^HistoIndex Pte Ltd, Singapore, Singapore; ^4^Department of Pathology, Peking University People's Hospital, Beijing, China

**Keywords:** nonalcoholic fatty liver disease, SHG/TPEF, steatosis, fibrosis, co-localization, animal model

## Abstract

**Background:**

Non-alcoholic fatty liver disease (NAFLD) is a global commonly occurring liver disease. However, its exact pathogenesis is not fully understood. The purpose of this study was to quantitatively evaluate the progression of steatosis and fibrosis by examining their distribution, morphology, and co-localization in NAFLD animal models.

**Methods:**

Six mouse NAFLD groups were established: (1) western diet (WD) group; (2) WD with fructose in drinking water (WDF) group; (3) WDF + carbon tetrachloride (CCl4) group, WDF plus intraperitoneal injection of CCl4; (4) high-fat diet (HFD) group, (5) HFD with fructose (HFDF) group; and (6) HFDF + CCl4 group, HFDF plus intraperitoneal injection of CCl4. Liver tissue specimens from NAFLD model mice were collected at different time points. All the tissues were serially sectioned for histological staining and second-harmonic generation (SHG)/two-photon excitation fluorescence imaging (TPEF) imaging. The progression of steatosis and fibrosis was analyzed using SHG/TPEF quantitative parameters with respect to the non-alcoholic steatohepatitis Clinical Research Network scoring system.

**Results:**

qSteatosis showed a good correlation with steatosis grade (*R*: 0.823–0.953, *p* < 0.05) and demonstrated high performance (area under the curve [AUC]: 0.617-1) in six mouse models. Based on their high correlation with histological scoring, qFibrosis containing four shared parameters (#LongStrPS, #ThinStrPS, #ThinStrPSAgg, and #LongStrPSDis) were selected to create a linear model that could accurately identify differences among fibrosis stages (AUC: 0.725-1). qFibrosis co-localized with macrosteatosis generally correlated better with histological scoring and had a higher AUC in six animal models (AUC: 0.846-1).

**Conclusion:**

Quantitative assessment using SHG/TPEF technology can be used to monitor different types of steatosis and fibrosis progression in NAFLD models. The collagen co-localized with macrosteatosis could better differentiate fibrosis progression and might aid in developing a more reliable and translatable fibrosis evaluation tool for animal models of NAFLD.

## 1. Introduction

Non-alcoholic fatty liver disease (NAFLD) is defined as the accumulation of fat in the liver without excessive alcohol consumption or other known liver pathologies. NAFLD encompasses a spectrum including “simple steatosis” (nonalcoholic fatty liver), non-alcoholic steatohepatitis (NASH), fibrosis, and cirrhosis or hepatocellular carcinoma (1–3). NAFLD is emerging as the most common type of chronic liver disease worldwide and might become the main indication for liver transplantation (4, 5). However, the exact pathogenesis and progression of NAFLD are not fully understood (6, 7), and NASH still lacks regulatory-approved pharmacotherapy (8, 9). Therefore, it is crucial to design effective interventions that can be safely used to treat NAFLD patients.

With the limitations in obtaining human samples and performing experimental drug studies in humans, NAFLD animal models are necessary to test the different pathogenic mechanisms and therapeutic targets of human NAFLD (10–13). Different murine models of NAFLD have been developed using diet, chemical induction, genetic modification, or a combination of these actions. However, at present, no other scoring system has been applied for histological evaluation, except for the Brunt or NASH Clinical Research Network (NASH CRN) scoring system (14, 15). This system is nonlinear and semiquantitative and cannot reflect the minor variability and extent of injury, especially for collagen changes. In addition, subjective evaluations using this system exhibited inter- and intra-observer variabilities. Consequently, precise, objective, and dynamic evaluation of liver histology in preclinical NAFLD animal models is valuable for developing more effective drugs.

The main histological features of NAFLD, including steatosis, inflammation, ballooning, and fibrosis, are often evaluated in preclinical models investigating novel therapies. Among the four features, steatosis is the most important diagnostic feature of NAFLD and is associated with progression to steatohepatitis and fibrosis (16, 17). Liver fibrosis is the most principal prognostic factor in NAFLD and is correlated with future liver-related events and long-term overall mortality (18–20). Accurate assessment of steatosis and fibrosis severity could contribute to the precision of drug efficacy evaluation, which is crucial for drug development for this disease.

Second-harmonic generation (SHG)/two-photon excitation fluorescence imaging (TPEF) techniques have been reported in the development of new algorithms to assess steatosis and fibrosis in the liver in the past decade (21–25). It is unclear whether this purely quantitative method can be used in different NAFLD mice models. In addition, unlike macrovesicular steatosis mainly observed in human NAFLD patients, macrovesicular and microvesicular steatosis often coexist in animal models. Therefore, developing a new objective evaluation system is particularly important for quantifying steatosis and fibrosis progression in preclinical NAFLD animal models. Rodent diets containing 45% kcal fat are very common, which are equally effective in promoting obesity. Fructose, which is recognized to promote hepatic *de novo* lipogenesis, lipid accumulation, and insulin resistance, typically results in steatohepatitis progressing to moderate fibrosis. Carbon tetrachloride (CCl4) enhances the effects of high-fat diet (HFD) and western diet (WD) on the rapid development of NASH and fibrosis (26–28). Therefore, this study focused on six different mice NAFLD models associated with diet, fructose, and CCl4 induction and developed an automated evaluation system by combining SHG/TPEF microscopy and an adaptive quantification algorithm to distinguish the progression of steatosis and fibrosis.

## 2. Materials and methods

### 2.1. Animals and diets

Male C57BL/6 mice were purchased from Beijing Vital River Laboratory Animal Technology Co. Ltd., China. They were maintained at 25°C with a 12 h light/dark cycle and allowed standard chow and water *ad libitum* until the time of the study. All protocols used in this study were approved by the Animal Experimental Ethical Committee of the Peking University People’s Hospital (No. 2019PHC004). WD (42% kcal fat, 42.7% kcal carbohydrate; WD; TD120528), and 45% HFD (45% kcal fat, 35% kcal carbohydrate; HFD; MD12032) were purchased from Medicience Ltd. (Jiangsu, China), and standard chow (18% kcal fat, 58% kcal carbohydrate) was provided by SPF Biotechnology Co., Ltd. (Beijing, China).

### 2.2. Mouse models of NAFLD

Eight-week-old male C57BL/6 mice were randomly divided into six NAFLD groups: (1) WD group; (2) WD with fructose (WDF) group, WD with a high sugar solution (23.1 g/L d-fructose and 18.9 g/L d-glucose) in drinking water; (3) WDF + carbon tetrachloride (CCl4) group, WDF plus intraperitoneal injection of CCl4 (10%, 2.5 μL/g of body weight), once a week; (4) HFD group; (5) HFD with fructose (HFDF) group, HFD with a high sugar solution (23.1 g/L d-fructose and 18.9 g/L d-glucose) in drinking water; and (6) HFDF + CCl4 group, HFDF plus intraperitoneal injection of CCl4 (10%, 2.5 μL/g of body weight) once a week. Mice remained on the experimental diets until sacrifice at 0, 4, 8, 12, 16 weeks (WD, WDF, WDF + CCl4 and HDF + CCl4 group), at 0, 4, 8, 12, 16, 24, and 32 weeks (HFD and HFDF group; *n* = 5 per timepoint) on diet to obtain specimens of different steatosis grades and fibrosis stage. Control mice were feed with normal diet after the experiment was initiated.

### 2.3. Liver histology analysis

The left lobe of the liver was paraffin-embedded, cut serially into 4-μm sections for direct SHG/TPEF imaging, and stained with hematoxylin and eosin and picrosirius red staining, as previously described (21). Slides were evaluated by a blinded expert liver pathologist for histologic features, including grades for steatosis and stages of fibrosis, according to the NASH CRN scoring system (15). In addition, the collagen proportional area (CPA), as determined by the % area stained with Sirius Red, was quantified from the histological images using ImageJ (National Institutes of Health, Bethesda, MD, United States) as per our standard procedures.

### 2.4. Image acquisition system

All images of the unstained tissue samples were acquired using a Genesis (HistoIndex Pte. Ltd., Singapore) SHG/TPEF imaging system. SHG microscopy was used to visualize collagen and steatosis structures were visualized using TPEF microscopy. The samples were excited with a 780 nm two-photon laser, the SHG signals were recorded at 390 nm, and the TPEF signals were recorded at 550 nm. Image tiles were acquired for each whole liver sample at 20 × magnification with a 512 × 512-pixel resolution per tile, and each tile had an actual dimension of 200 × 200 μm^2^. Multiple adjacent image tiles were captured to encompass the entire tissue area on each slide.

### 2.5. Establishment of qSteatosis and qFibrosis

Using the NASH CRN scoring system as the reference standard, automated measures of fibrosis and steatosis were developed, termed qSteatosis and qFibrosis, respectively. The sequential procedure for establishing the two indices comprised: (1) detection of fat vacuoles and collagen in different regions of the lobules, (2) quantification of defined architectural parameters that represented the characteristics of various histopathological features, (3) feature selection from the most correlated parameters, and (4) model construction, combining feature parameters into a single “signature” index for each of the two NASH histological components.

### 2.6. Feature detection and image quantification

#### 2.6.1. Steatosis

To evaluate the severity of steatosis, fat vacuole candidates were detected as black holes in the TPEF channel and then further identified as either macro-or micro fat vacuoles using the Classification and Regression Trees method (29) by examining the hole shape, cell structure, surrounding collagen, etc. The regions of steatosis consisted of not only fat vacuoles with high densities but also the hepatocytes around the vacuoles, which can be identified by image dilation in the binary image of fat vacuole detection. Similar to fibrosis quantification, dozens of steatosis parameters were extracted from the whole tissue image, as well as the central vein, portal tract, and perisinusoidal regions, to assess correlations with NASH CRN steatosis grades (22).

#### 2.6.2. Fibrosis

To evaluate the severity of liver fibrosis, collagen was detected in the SHG channels within the tissue area. Using Otsu’s automatic threshold method (30), the collagen signal was identified from background noise. Then, multiple feature parameters were extracted from the collagen signals, such as length, width, density, and intersections. As liver tissue can be divided into three histological regions, namely central vein, portal tract, and perisinusoidal, feature detection was also performed in each region. Together with the overall quantification, approximately 100 fibrosis parameters (22, 25) were extracted from each image to evaluate correlations with NASH CRN fibrosis stages.

#### 2.6.3. Co-localization

To evaluate the severity of fibrosis co-localized with steatosis, we evaluated fibrosis changes in the area close to the fat vacuoles in the perisinusoidal space. As steatosis was classified as macrosteatosis and microsteatosis, sub-analysis of fibrosis co-localized with each steatosis type was also performed by correlating co-localized fibrosis parameters with histological scoring.

### 2.7. Feature selection and model construction

For each animal model, fibrosis and steatosis parameters were correlated with the NASH CRN fibrosis stages and steatosis grades. The shared parameters, whose correlation ranked relatively high among these parameters in all animal models, were found with respect to fibrosis stages, steatosis grades, and time points for modeling. Linear models of these parameters were then created to identify differences between animals with different NASH scores.

### 2.8. Statistical analysis

The area under the receiver operating characteristic curve (AUC) was used to illustrate the performance of the scoring classifiers, built with single parameters or their linear combinations, as its discrimination threshold varied. The Spearman correlation test was used to correlate the parameters or their linear combinations with steatosis grades (0, 1, 2, 3) and fibrosis stages (0, 1, 2, 3, 4), and correlation coefficients were used to compare and select parameters and models. The statistical significance level was set at *p* < 0.05.

## 3. Results

### 3.1. Histology in different NAFLD mouse models

Histological examination (hematoxylin and eosin staining) demonstrated that macro- and micro-vesicular steatosis were clearly visible after 16 weeks in all six NAFLD models. The collagen pattern detected by SHG was highly consistent with that of light microscopic appearance and histochemical staining (Supplementary Figures 1, 2). No signs of stage 2 fibrosis were detected in WD, WDF, HFD, or HFDF mice until 24 weeks. However, stage 3 to 4 fibrosis was observed in the WDF + CCl4 and HFDF + CCl4 mice after 16 weeks. CCl4 treatment may accelerate HFD- and WD-induced steatohepatitis and fibrosis.

### 3.2. Good correlations were observed between the histopathology and SHG/TPEF assessments of steatosis in all mouse models

Liver steatosis progression was quantified by examining the fat vacuoles in the TPEF channel. Overall, 45 steatosis parameters were extracted from the whole-tissue images. Good correlations were observed for all steatosis parameters with steatosis scores in all six NAFLD mouse models (Table 1; Supplementary Figure 3). For representative parameters such as %Area (percentage of steatosis in the overall region), %MacroArea (percentage of macrosteatosis in the overall region), and %MicroArea (percentage of microsteatosis in the overall region), the Spearman correlations ranged from 0.823 to 0.953 (*p* < 0.05) in all mouse models (Supplementary Table 1). In addition, steatosis area parameters demonstrated high AUC values for differentiating steatosis grades (0.810-1).

**Table 1:** ROC analysis of the representative steatosis parameters in differentiating steatosis grades in the six mouse models.

Steatosis grade	WD			WDF			WDF + CCl4		
	%Area	%MacroArea	%MicroArea	%Area	%MacroArea	%MicroArea	%Area	%MacroArea	%MicroArea
0vs.123	1	1	0.953	NA	NA	NA	NA	NA	NA
01vs.23	NA	NA	NA	NA	NA	NA	0.965	0.983	0.632
012vs.3	1	0.990	0.899	1	1	1	0.967	0.892	0.617
Steatosis grade	HFD			HFDF			HFDF + CCl4		
	%Area	%MacroArea	%MicroArea	%Area	%MacroArea	%MicroArea	%Area	%MacroArea	%MicroArea
0vs.123	NA	NA	NA	NA	NA	NA	1	0.949	1
01vs.23	NA	NA	NA	1	1	0.932	1	0.921	0.905
012vs.3	1	1	1	0.893	0.810	1	1	0.857	0.964

ROC, receiver-operating-characteristics curve; WD, Western diet; WDF, WD + fructose; CCl4, carbon tetrachloride; HFD, high-fat diet; HFDF, HFD + fructose; NA, not available.

### 3.3. The qFibrosis index combining four shared morphological parameters faithfully recapitulated the fibrosis staging

Based on their quantitative trends of fibrosis stages and systemic AUC analyses, four shared parameters of collagen string (#LongStrPS, #ThinStrPS, #ThinStrPSAgg and #LongStrPSDis) were selected to combine qFibrosis indices, which showed good correlation with fibrosis stages in all animal models (*R*: 0.501–0.911, *p* < 0.05; Supplementary Figure 4; Supplementary Table 2). These indices can also accurately identify differences among fibrosis stages (AUC: 0.725-1; Table 2).

**Table 2:** Receiver-operating-characteristics curve analysis of the representative fibrosis parameters in differentiating fibrosis stages in six mouse models.

Fibrosis Stage	WD				WDF				WDF + CCl4			
	CPA	qFibrosis	qFibrosis-MacroCoLocalization	qFibrosis-MicroCoLocalization	CPA	qFibrosis	qFibrosis-MacroCoLocalization	qFibrosis-MicroCoLocalization	CPA	qFibrosis	qFibrosis-MacroCoLocalization	qFibrosis-MicroCoLocalization
0vs.1234	0.680	0.960	0.990	0.970	0.659	0.725	0.923	0.615	NA	NA	NA	NA
01vs.234	0.758	0.967	0.967	0.978	0.797	0.844	0.953	0.828	1	0.861	0.875	0.861
012vs.34	NA	NA	NA	NA	NA	NA	NA	NA	0.949	0.974	0.974	0.974
0123vs.4	NA	NA	NA	NA	NA	NA	NA	NA	0.760	1	1	1
Fibrosis Stage	HFD				HFDF				HFDF + CCl4			
	CPA	qFibrosis	qFibrosis-MacroCoLocalization	qFibrosis-MicroCoLocalization	CPA	qFibrosis	qFibrosis-MacroCoLocalization	qFibrosis-MicroCoLocalization	CPA	qFibrosis	qFibrosis-MacroCoLocalization	qFibrosis-MicroCoLocalization
0vs.1234	0.531	0.828	0.969	0.797	0.705	0.833	0.897	0.885	NA	NA	NA	NA
01vs.234	0.692	0.769	0.846	0.641	0.771	0.896	0.917	0.938	0.563	1	1	1
012vs.34	NA	NA	NA	NA	NA	NA	NA	NA	0.635	1	1	1
0123vs.4	NA	NA	NA	NA	NA	NA	NA	NA	0.854	0.938	0.917	0.938

CPA, collagen proportional area; ROC, receiver-operating-characteristics curve; WD, Western diet; WDF, WD + fructose; CCl4, carbon tetrachloride; HFD, high-fat diet; HFDF, HFD + fructose; NA, not available.

Furthermore, the performances of qFibrosis indices versus CPA for scoring fibrosis were evaluated using receiver operating characteristic analysis; these AUC values were mostly higher than those using CPA in differentiating fibrosis stages (0.725-1 vs. 0.531-1; Table 2).

### 3.4. qFibrosis co-localized with macrosteatosis showed superior performance compared to CPA, qFibrosis, and qFibrosis co-localized with microsteatosis in evaluating fibrosis severity

**Figure 1:**
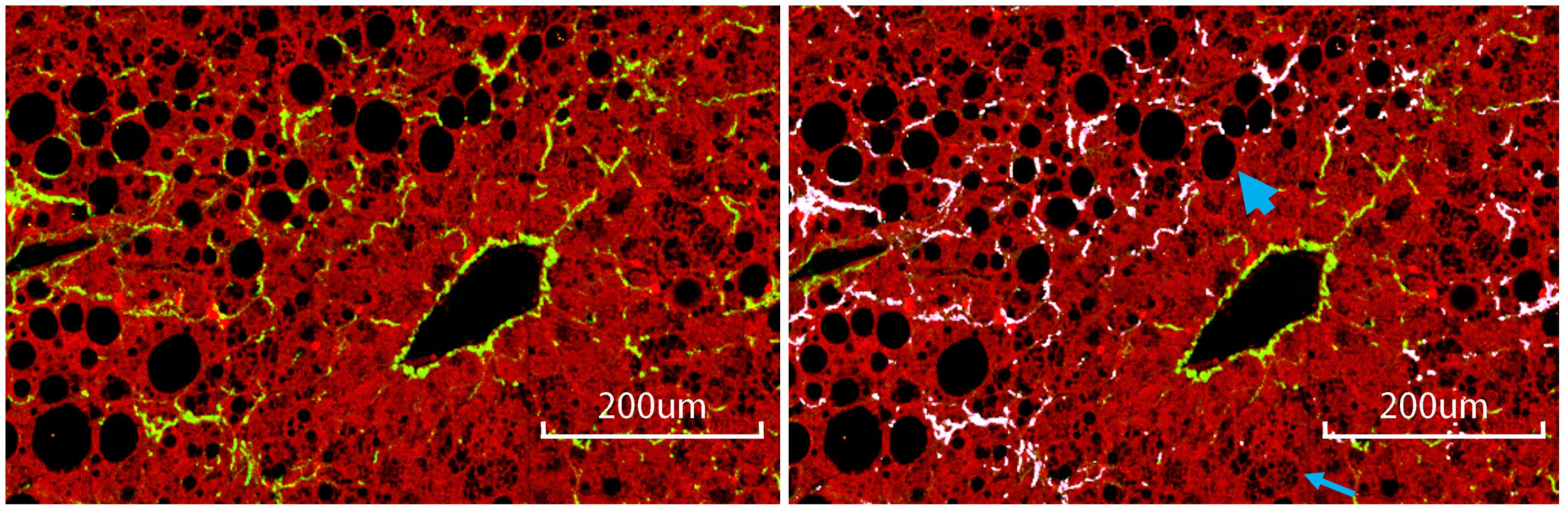
Collagen co-localized with steatosis. The subfigure on the left is a raw ROI image; the subfigure on the right is the corresponding annotated image, where white-colored collagen fibers are co-localized with steatosis. The bold and thin arrow points to an example of macro- and micro-steatosis, respectively. Scale bars, 200 μm.

The relationship between steatosis and fibrosis progression was further analyzed by examining the co-localization (Figure 1). The results revealed that qFibrosis co-localized with macrosteatosis was generally correlated better with histological scoring than qFibrosis (*R*: 0.598–0.914, *p* < 0.05), and qFibrosis co-localized with microsteatosis (*R*: 0.516–0.946, *p* < 0.05) in most animal models. Furthermore, using receiver operating characteristic analysis, the AUC values of qFibrosis co-localized with macrosteatosis for the detection of different stages were > 0.875 (AUC: 0.875–1), whereas the AUC values of qFibrosis were > 0.725 (AUC: 0.725–1) and the AUC values of qFibrosis co-localized with microsteatosis were > 0.615 (AUC: 0.615–1; Table 2).

## 4. Discussion

Animal models remain the best way to entirely study the pathophysiology of liver diseases and develop new treatments. However, few studies have analyzed the possibility of automated NAFLD evaluation using imaging techniques in animal models. In this study, we quantitatively evaluate the progression of steatosis and fibrosis in NAFLD animal models using SHG/TPEF imaging.

Hepatic steatosis is the key defining feature of NAFLD. Animal models often combine various degrees of macro-and microsteatosis. Some studies using animal models have analyzed new techniques that validate automated computerized systems for NAFLD diagnosis and staging. Ge et al. (31) first evaluated hepatic steatosis based on the quantitative digital analysis of Oil Red O in various mouse models and demonstrated that the area fraction was significantly associated with triglyceride deposition in the liver. Furthermore, Sethunath et al. (32) analyzed macro-and microsteatosis of fatty liver disease in murine models using machine-learning modalities. Ramot et al. (33) compared the manual semiquantitative microscope-based assessment with data output obtained from artificial intelligence software using a deep learning algorithm and showed an important association (*r* = 0.87, *p* < 0.01) among the semiquantitative methods performed by a human pathologist and automated diagnostic tools. Our results also showed good correlations between histopathology and SHG/TPEF-based steatosis parameters with steatosis scores in all our mouse models. This suggests that SHG/TPEF can be used to assess hepatic steatosis changes in different NAFLD mouse models.

The fibrosis stage is the main histological predictor of liver-specific outcomes. Accurate and quantitative evaluation of liver fibrosis in NAFLD is very important for predicting the risk of complications and tracking disease progression and should be considered as an effective endpoint in clinical trials of antifibrotic drugs. De Rudder et al. (34) demonstrated an automated method of measuring the total content of collagen in the picrosirius red-stained liver section regardless of collagen distribution. Roth et al. (35) demonstrated that INT-767 reduced collagen fiber signals using SHG imaging and suggested that SHG could be used to measure collagen improvements in preclinical NASH mice. Furthermore, based on the trends with respect to fibrosis stages and systemic AUC analyses, we selected qFibrosis by combining four parameters (#LongStrPS, #ThinStrPS, #ThinStrPSAgg, and #LongStrPSDis) to faithfully recapitulate the Ishak scores better than CPA. This suggests that the distribution, length, and width of collagen might be superior to total collagen quantitation and lay the foundation for the assessment liver fibrosis in NAFLD based on SHG microscopy.

In liver tissue with NASH, perisinusoidal fibrosis has a distinctive pattern and is most likely associated with the severity of steatosis (36). All four parameters for qFibrosis were derived from the perisinusoidal collagen. Therefore, we performed a co-localization analysis between qFibrosis and steatosis. Our results revealed that qFibrosis co-localized with macrosteatosis generally correlated better with histological scoring than the original qFibrosis and qFibrosis co-localized with microsteatosis in most animal models. Although macrosteatosis is mostly observed in patients with NAFLD, HFD-and WD-treated mice often show progression of macro-and microsteatosis (37, 38). Transitions and associations exist between macro- and micro-steatosis, and it has been hypothesized that large fat droplets are formed by the fusion of small droplets initially found on the surface of the endoplasmic reticulum (39, 40). Quantitative morphometric analyses revealed that INT-767 significantly reduced vesicular lipid droplet size, suggesting that macrovesicular steatosis is positively correlated with the severity of lobular inflammation and fibrosis (35). Therefore, qFibrosis co-localized with macrosteatosis could have more discriminative power to precisely reflect the dynamics of fibrosis progression in different NAFLD models.

All these NAFLD models have affirmed that SHG microscopy is an invaluable new platform to study and quantify steatosis and fibrosis, the key histological parameters of NASH. Although our SHG-based microscopy techniques allow for objective quantitative assessment of steatosis and fibrosis changes on a continuous scale, there are some limitations to be addressed. First, in this study, the animals in the WD, WDF, and HFD groups lacked grade 2 steatosis, which may affect the steatosis analysis, and the collection of liver samples at shorter time intervals (such as every 2 weeks) might be helpful to cover the whole steatosis grade spectrum. Second, in our study, fibrosis stages 1–4 were observed in liver tissues from WDF + CCl4 and HFDF + CCl4 animals, whereas only fibrosis stages 0–2 were covered by WD, WDF, HFD, and HFDF groups. It has been reported that without the acceleration from CCl4, milder liver fibrosis may only be triggered by a high-fat or high-fructose diet over a prolonged period (11, 12, 41). Third, MCD diet induced NAFLD models is not included. Although MCD diet could induce severe steatosis and obvious fibrosis in mice, there are several limitations, such as losing the weight of mice significantly, lacking insulin resistance, which were inconsistent with the clinical characteristics of NASH patients. Moreover, MCD diet mainly induced macrovesicular steatosis (42), which was different from HFD and WD diet treated mice.

In conclusion, quantitative assessment using stain-free SHG/TPEF technology could better differentiate fibrosis and steatosis progression in mouse models of NAFLD. Although further studies are required, we found that qFibrosis co-localized with macrosteatosis could be useful for the sensitive and specific monitoring of liver fibrosis changes in NAFLD mouse models. These results suggest that, in future studies, SHG/TPEF technology should be considered to improve the accuracy of histological assessment in NAFLD preclinical models.

## Data availability statement

The original contributions presented in the study are included in the article/Supplementary material, further inquiries can be directed to the corresponding author.

## Ethics statement

All protocols used in this study were approved by the Animal Experimental Ethical Committee of the Peking University People’s Hospital (No. 2019PHC004).

## Author contributions

FL, X-XW, XT, and H-YR designed the study. X-XW, RJ, and X-HL performed the experiments and supported the mouse experiments. F-FL, NW, and H-YR provided essential support and data interpretation. FL, X-XW, QY, and XT drafted the manuscript. All authors contributed to the article and approved the submitted version.

## Funding

This work was supported by grants from the National Natural Science Foundation of China (NSFC; 82170584 and 81870406) and the China National Science and Technology Major Project for Infectious Diseases Control during the 13th Five-Year Plan Period (2018ZX09201002-001-005).

## Conflict of interest

QY and XT are employed by HistoIndex or its subsidiary.

The remaining authors declare that the research was conducted in the absence of any commercial or financial relationships that could be construed as a potential conflict of interest.

## Publisher’s note

All claims expressed in this article are solely those of the authors and do not necessarily represent those of their affiliated organizations, or those of the publisher, the editors and the reviewers. Any product that may be evaluated in this article, or claim that may be made by its manufacturer, is not guaranteed or endorsed by the publisher.

## Abbreviations

AUC: area under the receiver operating characteristic curve
CCl4: carbon tetrachloride
CPA: collagen proportional area
HFD: high-fat diet
HFDF: HFD with fructose
NAFLD: non-alcoholic fatty liver disease
NASH: non-alcoholic steatohepatitis
NASH CRN: NASH Clinical Research Network
SHG/TPEF: second harmonic generation/two-photon fluorescence imaging
WD: western diet
WD: WD with fructose
