# Supplementary material for: Collagen co-localized with macrovesicular steatosis better differentiates fibrosis progression in non-alcoholic fatty liver disease mouse models
Source: Front Med (Lausanne). 2023 Jun 2;10:1172058. doi: 10.3389/fmed.2023.1172058 (PMC10272541; doi:10.3389/fmed.2023.1172058)
Supplement: Supplementary file 1 [file Data_Sheet_1.docx]

**Supplementary**

#### Supplementary Table 1. The representative steatosis parameters %Area, %MacroArea and %MicroArea correlated well with steatosis grades

| Parameter | WD | | WDF | | WDF+CCl4 | | HFD | | HFDF | | HFDF+CCl4 | |
| --- | --- | --- | --- | --- | --- | --- | --- | --- | --- | --- | --- | --- |
|  | *R* | *P* | *R* | *P* | *R* | *P* | *R* | *P* | *R* | *P* | *R* | *P* |
| %Area | 0.928 | 0.000 | 0.823 | 0.000 | 0.920 | 0.000 | 0.932 | 0.000 | 0.918 | 0.000 | 0.953 | 0.000 |
| %Macroarea | 0.922 | 0.000 | 0.823 | 0.000 | 0.869 | 0.000 | 0.896 | 0.000 | 0.877 | 0.000 | 0.859 | 0.000 |
| %Microarea | 0.844 | 0.000 | 0.823 | 0.000 | 0.591 | 0.001 | 0.932 | 0.000 | 0.930 | 0.000 | 0.904 | 0.000 |

Note: R is the Spearman correlation coefficient and P shows the statistical significance of the test)

**Supplementary Table 2. The representative fibrosis parameters qFibrosis, qFibrosis co-localized with macrosteatosis and qFibrosis co-localized with microsteatosis correlated better with fibrosis stages than CPA**

| **Parameter** | **WD** | | **WDF** | | **WDF+CCl4** | | **HFD** | | **HFDF** | | **HFDF+CCl4** | |
| --- | --- | --- | --- | --- | --- | --- | --- | --- | --- | --- | --- | --- |
|  | ***R*** | ***P*** | ***R*** | ***P*** | ***R*** | ***P*** | ***R*** | ***P*** | ***R*** | ***P*** | ***R*** | ***P*** |
| **CPA** | 0.387 | 0.092 | 0.388 | 0.091 | 0.748 | 0.000 | 0.374 | 0.189 | 0.411 | 0.080 | 0.325 | 0.219 |
| **qFibrosis**  **(Shared parameters)** | 0.703 | 0.001 | 0.501 | 0.024 | 0.931 | 0.000 | 0.576 | 0.031 | 0.617 | 0.005 | 0.911 | 0.000 |
| **qFibrosis-MacroCoLocalization** | 0.811 | 0.000 | 0.673 | 0.001 | 0.904 | 0.000 | 0.663 | 0.010 | 0.598 | 0.007 | 0.914 | 0.000 |
| **qFibrosis-MicroCoLocalization** | 0.690 | 0.001 | 0.516 | 0.020 | 0.892 | 0.000 | 0.600 | 0.023 | 0.663 | 0.002 | 0.946 | 0.000 |

Note: R is the Spearman correlation coefficient and P shows the statistical significance of the test)

**Figure Legends:**

**Supplementary Figure 1.** Representative pictures of H&E, PSR staining and SHG/TPEF image from mice of each group are shown at 8 weeks. (200×). Scale bars, 200µm; H&E, haemotoxylin and eosin; PSR, Picrosirius red; SHG, second harmonic generation; TPEF, two-photon excitation fluorescence.

**Supplementary Figure 2.** Representative pictures of H&E, PSR staining and SHG/TPEF image from mice of each group are shown at 16 weeks. (200×). Scale bars, 200µm; H&E, haemotoxylin and eosin; PSR, Picrosirius red; SHG, second harmonic generation; TPEF, two-photon excitation fluorescence.

**Supplementary Figure 3.** The trends of the representative steatosis parameters with respect to the steatosis grades from the six animal models. %Area, the percentage of steatosis at overall region; %MacroArea, the percentage of macrosteatosis at overall region; %MicroArea, the percentage of microsteatosis at overall region.

**Supplementary Figure 4.** The trends of qFibrosis (4 shared collagen parameters) along with the fibrosis stages from the six animal models. CCl4, carbon tetrachloride; #LongStrPS, Number of long strings at peri-sinusoidal region; #ThinStrPS, Number of thin strings at peri-sinusoidal region; # ThinStrPSAgg, Number of thin and aggregated strings at peri-sinusoidal region; #LongStrPSDis, Number of long and distributed strings at peri-sinusoidal region. The X-axis represents the stage of fibrosis, the Y-axis represents the number of various collagen strings in a unit area (1mm^2^).

**Supplementary Figure 5.** Representative images of fibrosis and steatosis co-localization at 8 weeks and 16 weeks from the six animal models, where purple-colored collagen fibers are co-localized with steatosis. the steatosis grade (S) and fibrosis stage (F) for the representative image has been added in the top right corner of each image.


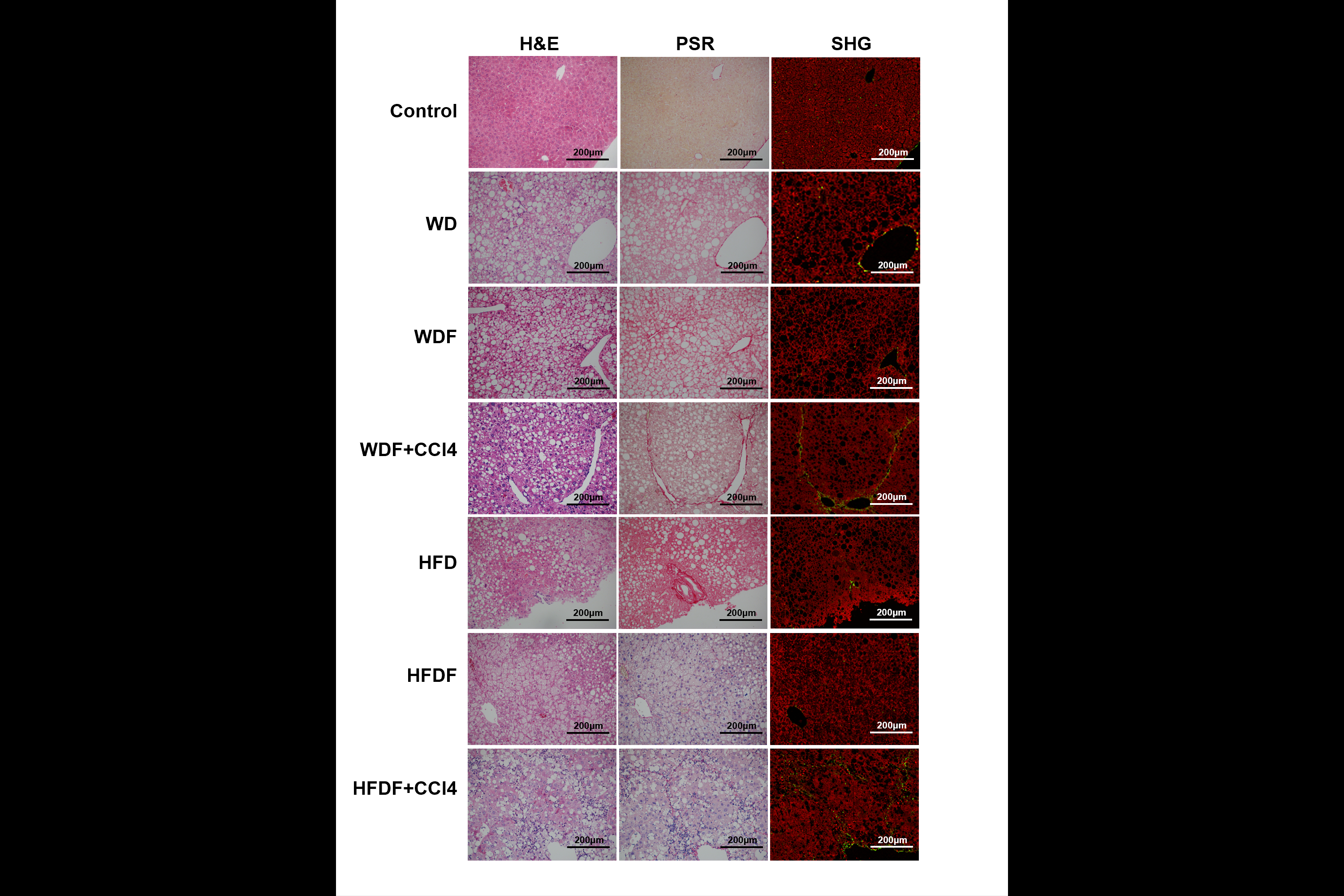


**Supplementary Figure 1.**


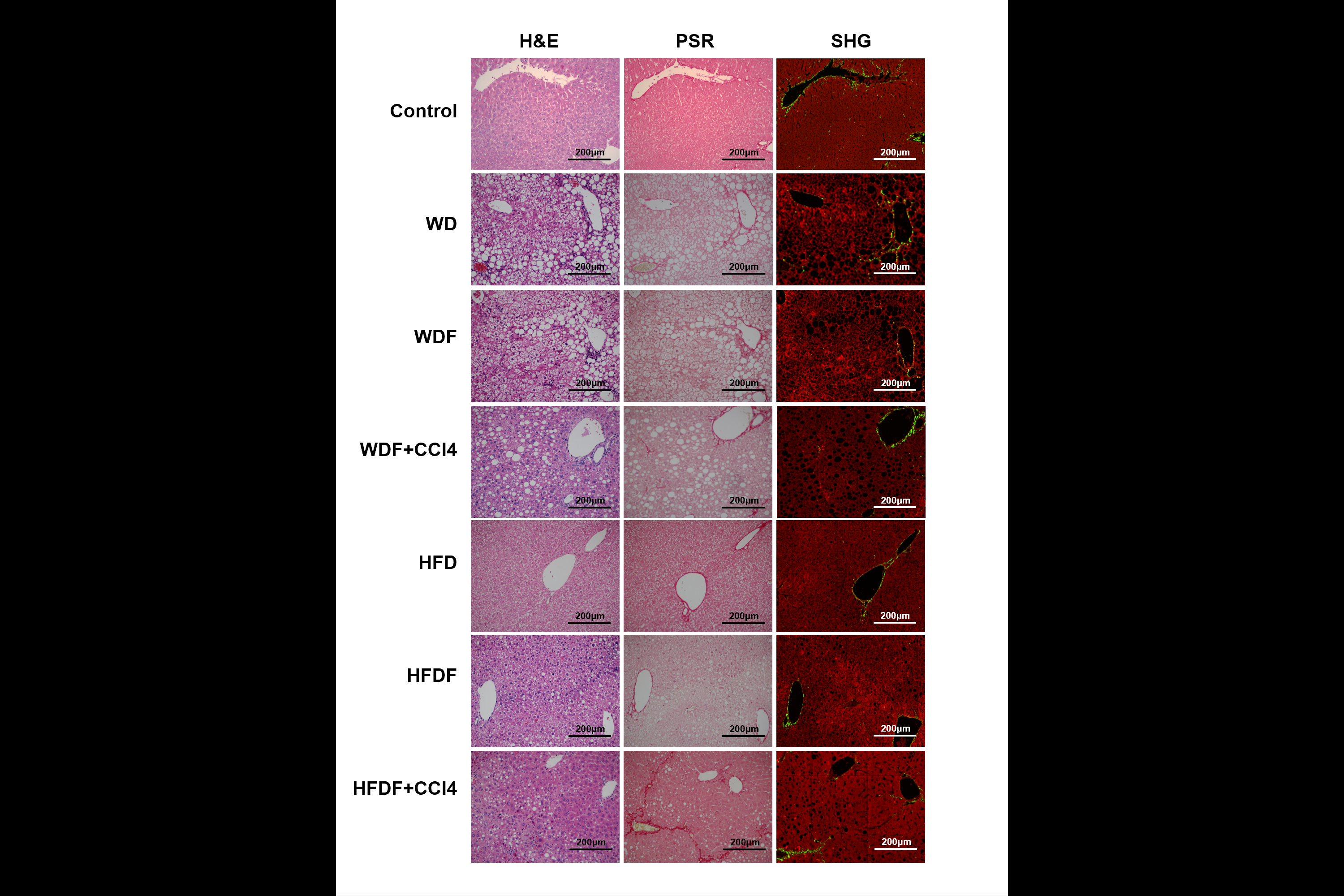


**Supplementary Figure 2.**


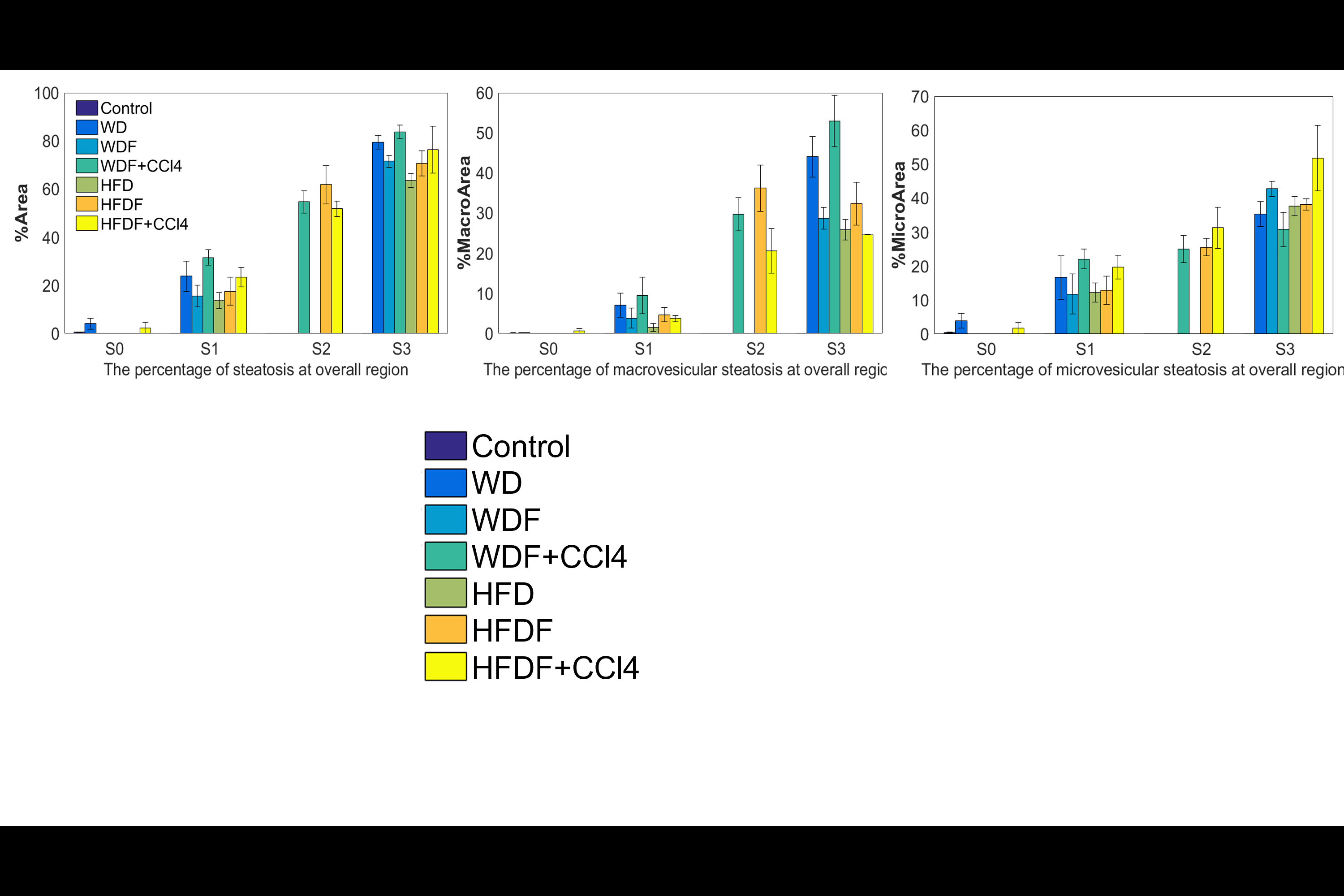


**Supplementary Figure 3.**


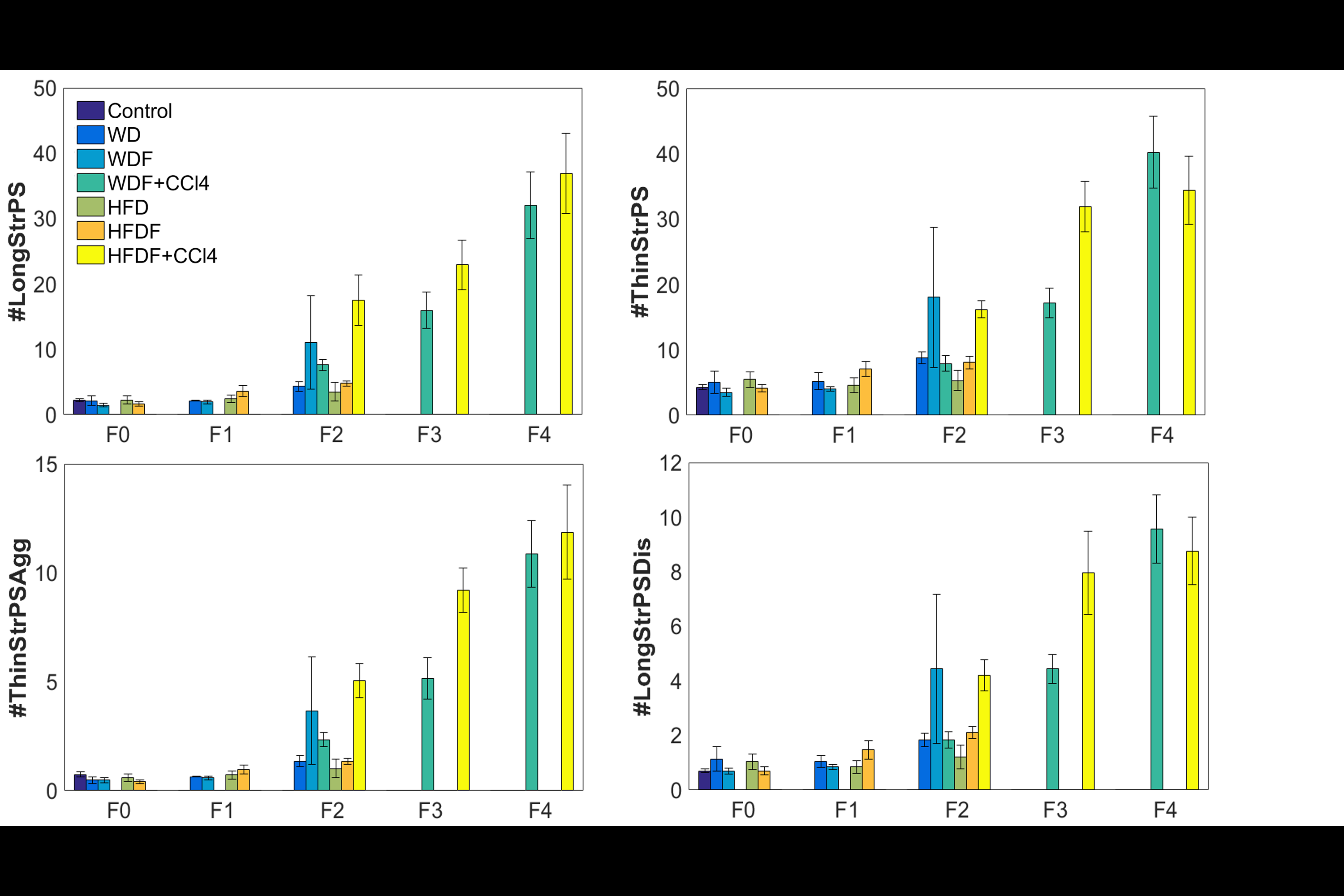


**Supplementary Figure 4.**


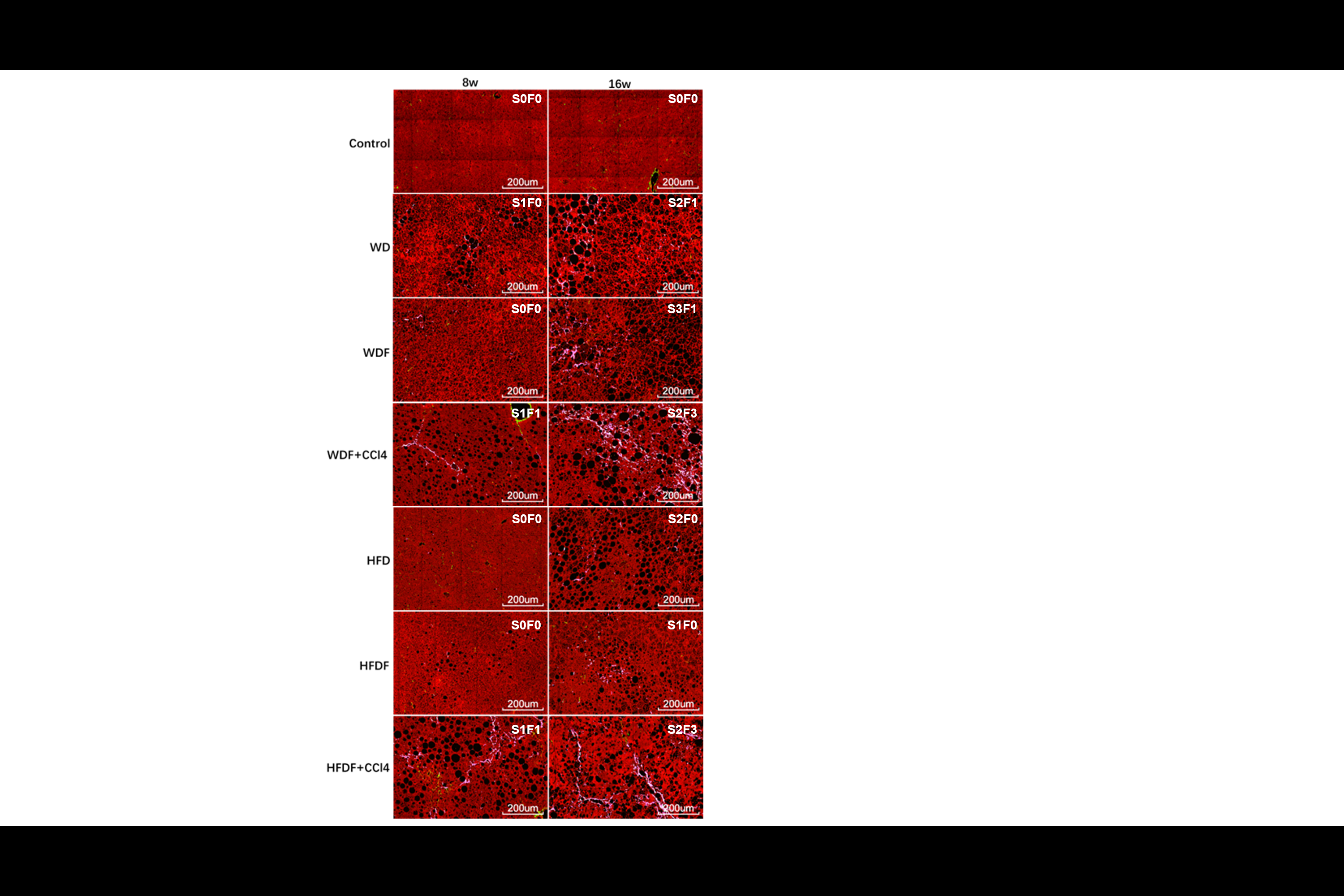


**Supplementary Figure 5.**
